# The Traditionally Amputated Uvula amongst Nigerians: Still an Ongoing Practice

**DOI:** 10.5402/2011/704924

**Published:** 2011-11-22

**Authors:** Adeyi A. Adoga, Tonga L. Nimkur

**Affiliations:** Department of Otolaryngology/Head and Neck Surgery, Jos University Teaching Hospital, PMB 2076, Plateau State, Jos, Nigeria

## Abstract

Traditional healers in Nigeria continue to perform uvulectomy for all throat problems despite the severe complications they present to physicians. It is a hospital-based prospective study done at the outpatient unit of the Department of Otolaryngology, Jos University Teaching Hospital, Jos, Nigeria to determine the prevalence of traditional uvulectomy, highlighting the dangers it portends with suggested ways of providing improved health outcomes for our people. We saw 517 new cases of which 165 (32%) patients aged 2 years to 53 years had their uvulae amputated consisting of 108 (65.5%) males and 57 (34.5%) females giving a male to female ratio of 2 : 1. One hundred and forty two (86.1%) patients had uvulectomy at childhood and 23 (13.9%) in adulthood. The commonest indication was throat pain (n = 36, 21.8%). The commonest complication was hemorrhage (n = 29, 17.6%). Forty six (27.9%) patients required hospital admission.

## 1. Introduction

For centuries, investigators have attributed various functions and conditions to the uvula, some speculative and others with definitive scientific basis [1]. Some have emphasized its influence in the tone of voice and others its immunological function [2]. Recently the role of the uvula in the moistening of the oropharyngeal mucosa has been described as evidenced by the experience of dryness of the throat following uvulopalatopharyngoplasty (UPPP) in patients [1, 2]. The uvula has been shown to secrete large amounts of thin serous saliva which bathes the oropharyngeal mucosa [1].

Traditional uvulectomy, a procedure which consists of cutting part or the entire uvula, is a common practice in sub-Saharan Africa. It is carried out by itinerant traditional surgeons who double as barbers using a sickle knife, performing other procedures such as incision and drainage of abscesses, circumcisions, and tooth extractions, and so forth [3–5]. The uvula is assumed to be the organ responsible for all throat conditions by these traditional surgeons, therefore, gets amputated.

Cutting equipments are not cleaned and/or sterilized, and there have been reported cases of use in upwards of 10 patients in a single session, therefore exposing individuals to complications such as hemorrhage, anemia, septicemia, tetanus, risk of the Human Immunodeficiency Virus (HIV) infection, and death [6–8].

These practices are still rampant and unchecked in our environment despite attempts made at discouraging it over the years.

This study aims to determine the prevalence of traditional uvulectomy in a North central Nigerian population and to further highlight the dangers this unwholesome practice portends with suggested ways of providing improved health outcomes for our people.

## 2. Methods

This is a hospital-based prospective study carried out at the outpatient unit of the Department of Otolaryngology, Jos University Teaching Hospital, Jos, Nigeria between the periods December 2009 and April 2011.

Following approval by the Ethical Clearance committee of the Jos University Teaching Hospital, all new patients seen within the study period with various otorhinolaryngological complaints were examined for the presence or absence of their uvula. Patients with amputated uvulae were further evaluated for the following.

1. Age at the time of amputation of the uvula.
2. The reason given at the time for amputating the uvula.
3. Complications following amputation of the uvula and if this required hospital admission.
4. Antibiotic regimen after uvulectomy.
5. Presence of initial indications for amputation.

## 3. Results

A total of 517 new cases were seen in the study period of which 165 (32%) patients had their uvulae amputated. These consisted of 108 (65.5%) males and 57 (34.5%) females giving a male to female ratio of 2 : 1. The age range of patients with amputated uvulae was 2 years to 53 years. Patients in the second decade of life constituted the largest group closely followed by those in the first decade (Table 1).

One hundred and forty two (86.1%) patients had uvulectomy at childhood and 23 (13.9%) had theirs in adulthood.

The indications for uvulectomy were throat pain (*n* = 36, 21.8%), cough (*n* = 4, 2.4%), dysphagia (*n* = 9, 5.5%), loss of appetite (*n* = 8, 4.8%), and a large majority of patients (*n* = 108, 65.5%) did not know the indication for the procedure being performed on them (Table 2).

**Figure 1:**
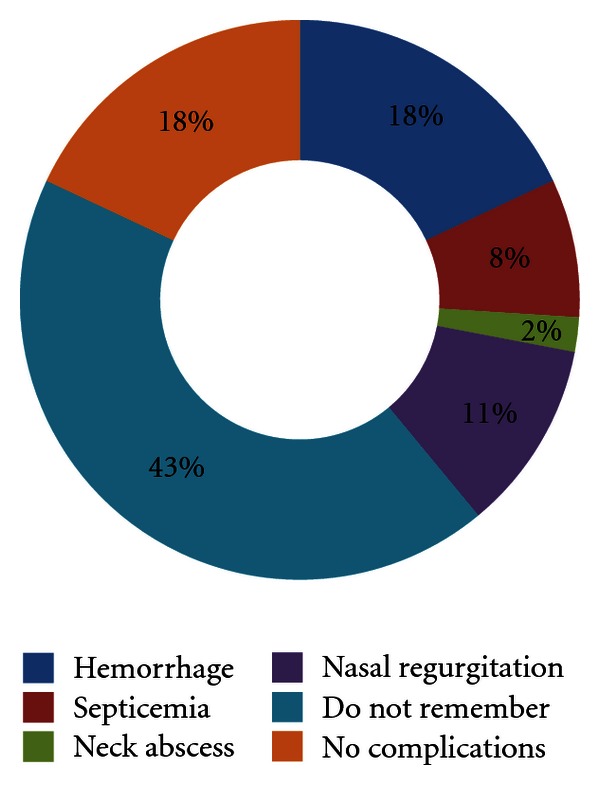
Types of complications following traditional uvulectomy.

The complications which followed uvulectomy in our series also varied, and they were hemorrhage (*n* = 29, 17.6%), septicemia (*n* = 14, 8.5%), neck abscess (*n* = 3, 1.8%), nasal regurgitation (*n* = 18, 10.9%). However, there were no complications in 30 (18.2%) patients and 71 (43%) patients do not remember if they had complications or not. Forty six (27.9%) patients required hospital admission for various complications (Figure 1).

Sixty one (37%) patients were not given antibiotics following uvulectomy, and 104 (63%) patients do not remember being given antibiotics or not following the procedure.

**Figure 2:**
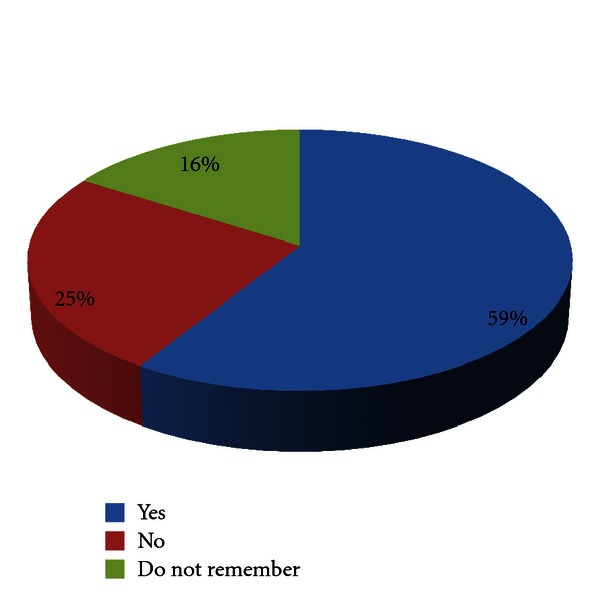
Patients with persistent symptoms following traditional uvulectomy.

Ninety seven (58.8%) patients still had the presence of the symptoms for which they had uvulectomy, 41 (24.8%) patients had cure for their symptoms, while 27 (16.4%) patients do not remember anything in this regard (Figure 2).

## 4. Discussion

Uvulectomy by traditional practitioners in Africa has been an age-long practice. It is a common procedure in Nigeria and several other African countries with documentations of this practice in countries outside Africa like Israel, Saudi Arabia, and some Middle Eastern countries [3–5, 9]. These practitioners are actually traditional healers or lay individuals who double as barbers performing their acts with a sickle knife and other unsterilized instruments [3, 4].

It is traditionally performed for infants and children in the first or second year of life with the belief that the elongated uvula is responsible for all throat problems including the suffocation of these children in their sleep [10]. In some parts of Africa, it is performed during certain ritual ceremonies and it involves the complete or partial removal of the uvula [11]. This study shows that uvulectomy was performed more for persons in the second decade of life and closely followed by those in the first decade. Majority of our patients had their uvula amputated in childhood in accordance with the reports that this procedure is commonly performed in children. That most of our patients do not remember the indications for the traditional uvulectomy they had or the complications which followed is a probable pointer to the fact that they had the procedure at childhood.

Other indications given for this procedure by the practitioners include sore throat, chronic cough, vomiting and diarrhea, rejection of breast milk by child, growth retardation, and fever [6, 12]. The commonest indication in our study is throat pain with other indications like dysphagia, cough, and loss of appetite recorded.

Some of these traditional practitioners' patients do well after the procedure but this unwholesome, unscientific, and unsupervised practice is potentially dangerous resulting in complications such as hemorrhage, anemia, septicemia, tetanus, risk of the Human Immunodeficiency Virus (HIV) infection, and death [6–8]. Hemorrhage is the commonest complication in our study, and 46 (27.9%) patients required hospital admission at various times for complications they developed from the procedure. Instruments used for the procedure are not sterilized, and there are reports of use of these instruments on several patients in the same session thereby exposing them to untoward complications some of which have been established in time past including the Human Immunodeficiency virus (HIV) infection and hepatitis with even newer complications reported recently such as hemorrhage from the tonsils and cavernous sinus thrombosis [13, 14]. In cases where uvulectomy is performed in the absence of adenoids, velopharyngeal incompetence may result with nasal regurgitation of meals as seen in a number of patients in our series. Almost 40% of the patients in this study were not given antibiotics following the procedure, and it can be deduced that even those who do not recollect the events following the procedure were not given antibiotics as well, hence the development of complications such as neck infections and septicemia started.

Over half of our patients still had the presence of the symptoms which ab initio was given as indication for traditional uvulectomy. This is because these practitioners have no knowledge of the anatomy and physiology of the structures in the pharynx with the resultant amputation of the uvula for other etiological factors responsible for pharyngeal symptoms. There have been reports of uvulectomy performed in patients who had recurrent tonsillitis because they presented with throat symptoms and of course their symptoms persisted despite the amputation of their uvulae [12].

In the developed world, uvulectomy is done as part of a combined procedure uvulopalatopharyngoplasty, to ameliorate conditions like snoring [15]. There is not a mention in the literature from these parts of the world of amputation of the uvula on account of the indications listed for traditional uvulectomy.

The dangers associated with traditional uvulectomy have been enumerated; the issue to address is the mode of improving the health care outcomes for the people in our society. The following suggestions may very well be of help; a well-designed health education program at all levels of society should be embarked upon in which the traditional practitioners and the would-be “uvulectomy” are adequately educated on the dangers of this practice. These traditional practitioners claim there are no risks associated with this practice and the likelihood is not giving up; therefore, a close collaborative program of training and retraining them is more likely to be effective in reducing the morbidity associated with their work and overall improving the health outcome for patients.

## 5. Conclusions

Traditional uvulectomy is still being rampantly practiced in our society. It should be out rightly discouraged and laws promulgated to stop it or programs for training and re-training of the practitioners instituted to improve their practice and safeguard people in our society.

## Figures and Tables

**Table 1:** Age distribution of patients presenting with amputated uvulae.

Age group (years)	Frequency	Percentage
0–10	68	41.2
11–20	74	44.8
21–30	13	7.9
31–40	9	5.5
41–50	0	0
51–60	1	0.6

Total	165	100

**Table 2:** Indications for traditional uvulectomy.

Indications	Frequency	Percentage
Throat pain	36	21.8
Cough	4	2.4
Dysphagia	9	5.5
Loss of appetite	8	4.8
Do not remember	108	65.5
